# Comparative sanitation data from high-frequency phone surveys across 3 countries

**DOI:** 10.1016/j.dib.2024.110635

**Published:** 2024-06-13

**Authors:** Amy R. Lewis, Andrew R. Bell, Ana Casas, Beata Kupiec-Teahan, José Mendoza Sanchez, Simon Willcock, Fiona Anciano, Dani J. Barrington, Mmeli Dube, Paul Hutchings, Caroline Karani, Arturo Llaxacondor, Hellen López, Anna L. Mdee, Alesia D. Ofori, Joy N. Riungu, Kory C. Russel, Alison H. Parker

**Affiliations:** a School of Environmental & Natural Sciences, Bangor University, Bangor, Gwynedd LL57 2DG, UK; b Department of Global Development, Cornell University, Ithaca, NY 14850, USA; c School of Water, Energy and Environment, Cranfield University, Cranfield MK43 0AL, UK; d Bangor Business School, Bangor University, Bangor, Gwynedd LL57 2DG, UK; e Department of Management Science, Pontificia Universidad Católica del Perú, Perú; f Instituto de Estudios Peruanos, Perú; g Net Zero and Resilient Farming, Rothamsted Research, Harpenden, Hertfordshire AL5 2JQ, UK; h Department of Political Studies, University of the Western Cape, South Africa; i School of Population and Global Health, The University of Western Australia, 35 Stirling Highway, Crawley 6009, Western Australia, Australia; j School of Civil Engineering, University of Leeds, LS29JT, UK; k Meru University of Science and Technology, 972-60200, Kenya; l Sanima, Av. Grau 629, Barranco, Lima, Perú; m School of Politics and International Studies, University of Leeds, LS29JT, UK; n Department of Landscape Architecture and the Environmental Studies Program, University of Oregon, Eugene, OR 97403, USA

**Keywords:** Sanitation, Water Supply, Container-based Sanitation, Off-grid Sanitation, WASH, Smartphone Survey, Wellbeing, Poverty

## Abstract

With less than half of the worldʼs urban population having safely managed sanitation due to the high cost and difficulty of building sewers and treatment plants, many rely on off-grid options like pit latrines and septic tanks, which are hard to empty and often lead to illegal waste dumping; this research focuses on container-based sanitation (CBS) as an emerging off-grid solution. Off-grid sanitation refers to waste management systems that operate independently of centralized infrastructure and CBS is a service providing toilets that collect human waste in sealable containers, which are regularly emptied and safely disposed of. These data relate to a project investigating CBS in Kenya, Peru, and South Africa, focusing on how different user groups access and utilize sanitation – contrasting CBS with other types. Participants, acting as citizen scientists, collected confidential data through a dedicated smartphone app designed by the authors and external contractors. This project aimed to explore the effective scaling, management, and regulation of off-grid sanitation systems, relevant to academics in urban planning, water and sanitation services, institutional capability, policy and governance, and those addressing inequality and poverty reduction.

The 12-month data collection period offered participants small incentives for weekly engagement, in a micro payment for micro tasks approach. Participants were randomly selected, attended a training workshop, and (where needed) were given a smartphone which they could keep at the end of the project. We conducted weekly smartphone surveys in over 300 households across informal settlements. These surveys aimed to understand human-environment interactions by capturing daily life, wellbeing, income, infrastructural service use, and socioeconomic variables at a weekly resolution, contributing to more informed analyses and decision-making.

The smartphone-based approach offers efficient, cost-effective, and flexible data collection, enabling extensive geographical coverage, broad subject areas, and frequent engagement. The Open Data Kit (ODK) tools were used to support data collection in the resource-constrained environment with limited or intermittent connectivity.

Specifications Table

**Table:** 

Subject	Waste Management and Disposal
Specific subject area	Water, Sanitation and Health Sciences.
Type of data	Table, Documents, Chart, Graph, Figure, Processed
Data collection	Questionnaires were based on World Health Organisation/UNICEF standardised metrics for wellbeing and sanitation. We used Open Data Kit (ODK) to allow participants to self-report data on smartphones. Over 300 participants were randomly selected and trained to use the survey. The survey was live for over 80 weeks to collect weekly surveys over a period >12-months. Variables collected also included data on water access, expenditure, livelihood or environmental shocks experienced, electrical connections, and elections. The number of questions per task ranged from 1 question to 80 (mean = 20, skip logic was employed to reduce the burden. The majority of tasks could be completed between 3 and 20 min. Bonus points were used to incentivise completion of additional tasks within each survey. Task completion was not compulsory and intended to be completed in participants free time. Participants were reimbursed through data and talk time on a weekly basis depending on number of tasks completed. Participants were able to keep the project smartphone, or if using their own, receive
	higher compensation. Finalised data sets were anonymised, and Geo locations removed from the data sets. We have provided 16 final data sets in the dataset folder. We also have provided 3500 surveys, which were rolled out across three countries which were available to complete over 80 weeks. We provide 21 files which were used to calculate points for participants and data cleaning code files. In addition, we have provided sampling strategies and training materials.
Data source location	Nairobi, Kenya (1.2921° S, 36.8219° E), Cape Town, South Africa (33.9221° S, 18.4231° E), Lima, Peru (12.0464° S, 77.0428° W)
Data accessibility	Repository name: UK Data Service ReShare Data identification number: http://doi.org/10.5255/UKDA-SN-857073 Direct URL to data: https://reshare.ukdataservice.ac.uk/857073/

## 1 Value of the Data

• The cost and logistic challenges of engaging respondents, coupled with the sheer volume of different topics covered, and variation in respondents’ capacity and willingness to answer, mean that a social data collection campaign may be extensive in geography, broad across subject areas, and frequent in engagement – but typically not more than one of these at any one time. Smartphone surveys can be used to tackle all these issues simultaneously, yielding rich data addressing both temporal and spatial context required to answer complex research objectives.
• We conducted weekly, self-administered, smartphone surveys in over 300 households living in difficult to reach informal settlements in Kenya, South Africa, and Peru over a 12-month period. This granularity provides a comprehensive view, contributing to more informed analyses and decision-making.
• This dataset can be used to analyse and compare sanitation systems, including container-based sanitation (CBS) and other types, across different user across the countries. It provides weekly insights into daily life, wellbeing, income, infrastructural service use, and socioeconomic variables in households and can inform decisions on scaling, managing, and regulating off-grid sanitation systems.
• Researchers can reuse this dataset [1] to explore different aspects of daily life, potentially uncovering patterns and trends that contribute to a deeper comprehension of the dynamics of relationships between humans and their environment.

## 2 Background

To protect human health and the environment, sanitation systems must separate people from their excreta and treat it. This involves not just technology but also finance, government policies, and human behaviour. Sewers and wastewater treatment plants can provide safe sanitation but are expensive and difficult to build, especially in dense urban areas or where land ownership is unclear. Only 45 % of the world's urban population has safely managed sanitation, with many using off-grid options like pit latrines and septic tanks [2]. These are hard to empty in densely populated, sloped, or high water table areas and often lead to illegal waste dumping in water sources. Effective off-grid solutions require proper waste collection, treatment, and disposal. This research focuses on container-based sanitation (CBS), an emerging off-grid option. The Container Based Sanitation Alliance (CBSA) defines CBS as “a sanitation service which provides toilets that collect human excreta in sealable, removable containers on a regular basis and safely disposes of or reuses excreta” [3].

Through the Scaling up Off-Grid Sanitation project (ES/T007877/1), we studied off-grid sanitation in Kenya, Peru, and South Africa, focusing on how different respondents access and utilize Container-Based Sanitation (CBS). The objective of this research project is to expand knowledge by challenging the traditional assumptions of urban sanitation planning that focus on on-grid systems. It aims to explore how off-grid sanitation systems can be scaled up, managed, and regulated effectively. This research is relevant to academics in urban planning, water and sanitation services, institutional capability, policy and governance, and those working on basic service provision to address inequality and poverty reduction.

Participants acted as citizen scientists and collected confidential data through a dedicated smartphone application. The project aimed to identify barriers to implementation and investigated use of CBS, sharing insights with other municipal governments and CBS companies interested in implementing container-based sanitation for off-grid areas. The 12-month smartphone survey offered participants small incentives for weekly engagement, attendance at a training workshop, and access to a smartphone. We conducted the survey at weekly resolution in over 300 households in informal settlements in Kenya, South Africa, and Peru over a 12-month period. This smartphone survey aimed to understand human-environment interactions by capturing daily life, wellbeing, income, infrastructural service use, and socioeconomic variables at a weekly resolution. We also collected weekly data on livelihood or environmental shocks. Shocks are sudden, acute events with immediate impacts, disrupting normal functioning and leading to rapid changes in a system, such as natural disasters or economic crises [4].

Traditional data collection models are constrained by cost, logistics, and respondent limitations. However, the widespread use of mobile phones, particularly in diverse socio-economic contexts, offers a solution to the aforementioned problems. The authors propose leveraging mobile technology for frequent, short engagements with respondents, overcoming previous barriers.

## 3 Data Description

**This folder contains:**

1. **Folder: SOS_Consent and training materials**
  I. SOS_Permits
  II. Privacy Policy for Data exchange.docx
    Privacy policy for using our Data Exchange app.
  III. SOS_Consent_form.docx
    Participant consent form in English, used across all countries.
  IV. SOS Ethics approval letter March 2021.docx
    Ethical approval letter from Bangor University.
  V. SOS_Phone_setup_documentation.docx
    Documentation describing the app installation and download of the ODK surveys onto a phone.
  VI. SOS_ Survey_Design_References.docx
    Table describing key indexes or standardised questionnaires within the survey with references.
  VII. SOS_Training_2021_english.pptx
  VIII. Training materials provided to the in-country teams to assist with training workshops.
2. **Folder: SOS_Datasets**
  I. SOS_Consolidated Users.xlsx
    Consolidated final users within the SOS project and finalised according to the SOS_Cleaning_Protocol in section 3.i below. This is the master data set allowing the link between demographic variables, as well as country and CBS user type to the ID_Key. This ID is unique to an individual, and all data sets are linked to this ID.
    All data sets below have been cleaned and anonymised according to the SOS_Cleaning protocol.docx in section 3.i below. All data for all countries have been merged into a single file by task topic. All variable names relate to the ODK codes which can be found in Folder 4. This also describes the data types (e.g., single, or multiple choice).
  II. SOS_Demographics Monthly.xlsx
    Key expenditures by group (e.g., rent, food, travel) plus monthly GPS check ins.
  III. SOS_Demographics Weekly .xlsx
    Key expenditures by group (e.g., rent, food, travel).
  IV. SOS_Elections.xlsx
    Data from questions related to upcoming elections and any sanitation specific changes because of them.
  V. SOS_Electricity.xlsx
    Data on electricity supply and disruptions experienced.
  VI. SOS_Poverty_likelihoods_Kenya.xlsx
    Uses an index from Innovations for Poverty Action [5]
  VII. SOS_Poverty_likelihoods_Peru.xlsx
    Uses an index from Innovations for Poverty Action [5]
  VIII. SOS_Poverty_likelihoods_SA.xlsx
    Uses an index from Innovations for Poverty Action [5]
  IX. SOS_Sanitation Access.xlsx
    Data regarding an individual's sanitation access and servicing.
  X. SOS_Shocks.xlsx
    Captures if a shock was experienced, the shock category (e.g., livelihood, health, or weather) as well as self-reported impacts on the individual.
  XI. SOS_SNA.xlsx
    Captures social network of Container Based Sanitation (CBS) users.
  XII. SOS_WASH Monthly.xlsx
    All WASH modules use standardised classifications for toilet systems and water points [6]. The monthly WASH survey reports if a complaint was registered, and if was dealt with over the last month.
  XIII. SOS_WASH Once.xlsx
    These data are related to one off question describing the toilet within the household.
  XIV. SOS_WASH Quarterly.xlsx
    These data relate to an individual's likelihood to refer CBS toilets to others and captures what an ideal toilet means to them.
  XV. SOS_WASH Weekly.xlsx
    A weekly survey capturing the access and barriers to sanitation and water (for drinking, cooking, sanitation and washing).
  XVI. SOS_Wellbeing.xlsx
    We used multiple metrics of wellbeing including World health organisation (WHO-5) index [7] as well as sanitation specific wellbeing questions as developed by Ross (2021) [8]. As we expected wellbeing to change regularly, these data did not contain skip logic.
3. **Folder: SOS_Survey release dates and download code**
  I. SOS_Cleaning protocol.docx
    Comprehensive document describing the cleaning protocol for all data sets in Section 2.
  II. SOS_Compensation_Calculator.xlsx
    Calculator used to calculate scores per task that each respondent could receive if they were using their own phone or a project phone. This was localised by country and dependant on phone and data costs.
  III. SOS_Points_assignment.R
    R code Assigning points to individuals based on weekly scores.
  IV. SOS_Server data download.R
    R code accessing the central server (now deleted) and merging the points scored by everyone by each week.
  V. SOS_Survey_release_dates.xlsx
    An excel file listing the names of each ODK form (see all forms in section 4) and their release dates and points value. Baseline surveys we available during the whole survey period to ensure completion, weekly surveys open for 7 calendar days. Some questionnaires had additional bonus tasks for which responded were awarded extra points.
  VI. SOS_Total_Form.list.xlsx
    List of all 3022 forms uploaded to the ODK central server.
  VII. SOS_Python Code (Folder of 15 files).
    This Folder contains 15 Python code files which clean each of the individual data sets. Each file is named according to the data set in question; for example, Python_code_Wellbeing_Dataset.ipynb is the code used to clean the wellbeing data sets.
4. **Folder: SOS_Survey_ODK_code**
  This folder contains all surveys uploaded to the Open Data Kit (ODK) central server. Each ODK form is attached to a “prefill.xlsx” file. This contains information on the points and longevity of each form. Within the ODK form itself there are three tabs “Survey”, “choices” and “settings”. The survey tab is all possible questions that the respondent could answer. See “label::en” for English questions and “label::XX” for alternative languages. Each question is related to a single variable “name”, regardless of the language the question was formulated in. Column header “type” refers to the type of question, this may be free text, multiple choice, or GPS position. See https://getodk.org/ for all code interpretations. Skip logic was used within these surveys. The “relevant” column contains all skip logic related to that variable and references other previously mentioned variables.
  The choices tab relates to all multiple or single choice options available to the respondents. Although, these may have been numerically coded, but were presented to the respondent in one of two survey languages. All value labels for individual variables/questions can be found in this tab. The Settings tab states the form title and allows you to set the default language.
  Within each country we had slight variations in the questions asked to ensure they were contextually relevant (except where we were following international indexes- see folder 1.iv SOS_ Survey_Design_References.docx). Container Based Sanitation (CBS) users were asked different questions compared to non-users (NU, i.e., those using other forms of sanitation). Therefore, for each country we had separate surveys: CBS or NU.
  I. Kenya > English & Swahili
  II. Peru > English & Spanish
  III. South Africa > English & Xhosa

## 4 Experimental Design, Materials and Methods

### 4.1 Project overview

**Fig. 1:**
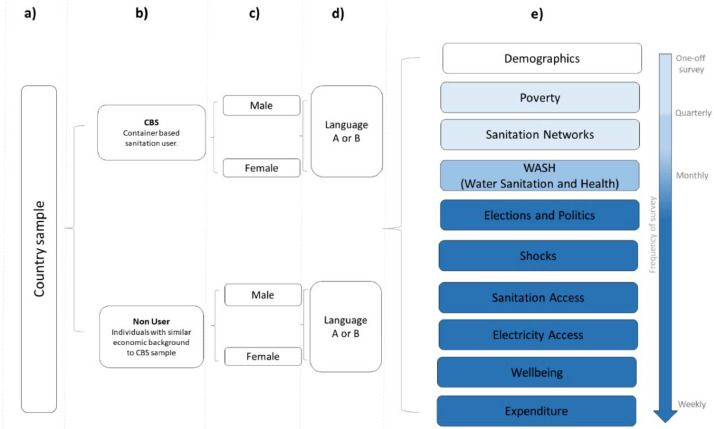
Overview of the sample and survey design, which was conducted in three countries: Peru, South Africa, and Kenya. Within each country we conducted high frequency surveys with approximately 100 individuals (a). The sampling aimed to match ∼50 users and ∼50 non-users of container-based sanitation products (CBS) (b). The sampling also intended to have a mix of male and female users and non-users (c). Each individual was able to answer the questionnaires in their preferred language (d) which was the prevalent language of the country or English. This meant that each questionnaire had been translated and stored on the server in multiple languages. Section e) describes the key tasks that respondents were asked to complete. These ranged from a one-off survey as well as quarterly, monthly, or weekly surveys as shown by the colour strength. The questionnaire only varied at two levels by a) country b) user-type, to ensure the survey was relevant in each context.

This work took place under the ‘Scaling-up Off-grid Sanitation’ project (SOS; ES/T007877/1), and we outline below the methods used to collect the smartphone survey work package of this project. The project was piloted in four countries: Haiti, Peru, Kenya, and South Africa. See Fig. 1 for an overview of the sampling design and the key tasks completed by respondents at various frequencies. The final project was only rolled out in three countries: Peru, South Africa, and Kenya.

**Fig. 2:**
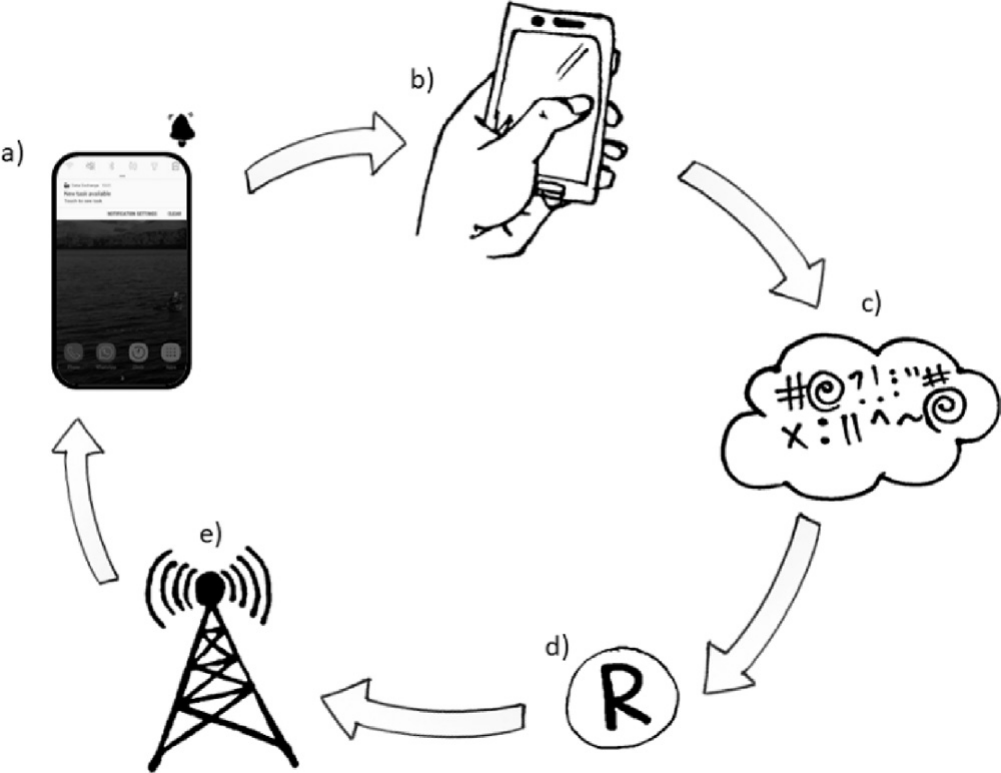
The Data exchange app[10] and wider system showing a) the interface giving notifications and a filtered list of micro tasks to complete. b) Participants are taken to the correct task in ODK. c) once completed forms are encrypted & sent to a server when there is a data connection d) Data are scraped from server at regular intervals to calculate the “top ups” due to participants using an R package[11]. e) Top ups values are sent to mobile providers and sent directly to participants devices as compensation. Illustrations by A.R.L.

We deployed high frequency phone surveys using ODK [9] software over a yearlong study. In exchange for phone ownership, data and talk time, we administered weekly, short questionnaires using an app called Data Exchange [2]. See Fig. 2 for an illustration of the Data Exchange app and wider system. The Data Exchange app allowed us to use push notifications to our respondents to remind them a new task was available to complete, it indicated how long it may take, and how many points they could score (see the compensation section below for a detail of the values). All participation was voluntary. Once the respondent had selected a task that was available, they were taken to the ODK task (key tasks are illustrated in Fig. 1, and details of each task are described in Table 1 below). Once completed, the survey was encrypted and stored on a cloud server hosted by ODK [9] (Fig. 2c). Weekly downloading of the data enabled us to quickly analyse the number of points scored per respondent using RuODK [10] an R software script for connecting to an ODK server (Fig. 2d). Points were converted to local currencies then calculated across the week per individual. These final weekly scores were then sent back to the field teams that then topped the participants up with mobile money or talk time.

**Table 1:** Number of points scored by each survey element, as repeated throughout the year.

Form name	Repeats over the year	Basic number of points available	Bonus points available	Total possible points per task	Total Points Across Year
Demographics-BASELINE	1	15	5	20	20
Demographics [Expenditure]- WEEKLY	40	5		5	200
Demographics [Expenditure]- - MONTHLY	12	10		10	120
Election	52	5		5	260
Electricity	52	5		5	260
Household (Poverty)	4	10	10	20	80
Journey_task_cbs_collection	1	10		10	10
Journey_task_community_toilet	1	10		10	10
Journey_task_truck_road	1	10		10	10
Sanitation_access_cbs_users / non users	52	10	5	15	780
Shocks	52	10	10	20	1040
SNA_CBS_users baseline (plus quarterly)	1	15		15	15
SNA quarterly	4	5		5	20
Wash_monthly	12	5		5	60
Wash_once	1	15		15	15
Wash_quarterly	4	10	5	15	60
Wash_weekly	52	10	10	20	1040
Wellbeing	52	10		10	520
TOTAL					4520

### 4.2 Ethics and compensation

Overall ethical approval for the project was approved by Bangor University, UK, College of Environmental Sciences and Engineering Ethics Committee Approval Number: COESE2021SW01A. Team leads in all countries obtained ethical approval from relevant bodies (see the SOS_Permits folder in repository 1) and informed consent was obtained from all survey participants to take part in the studies. An example consent form is available in Folder 1 in the repository.

Compensation was based on the approved project budget but was also dependent on the average cost of mobile phones purchased by the project. We calculated the value of points that could be achieved throughout the year if a respondent filled all tasks and bonus sections within the surveys (see Table 1).

All respondents received the same overall compensation relative to their level of engagement within each country. Respondents were made aware of how much they could score during their workshop training. Compensation was solely based on responses to tasks, we asked questions such as “did anything change since you last spoke to us about this” a “yes” or “no” response would receive the same value in compensation. Bonus points were available to those that we requested to define a changed state “would you like to tell us a bit more about this”.

**Fig. 3:**
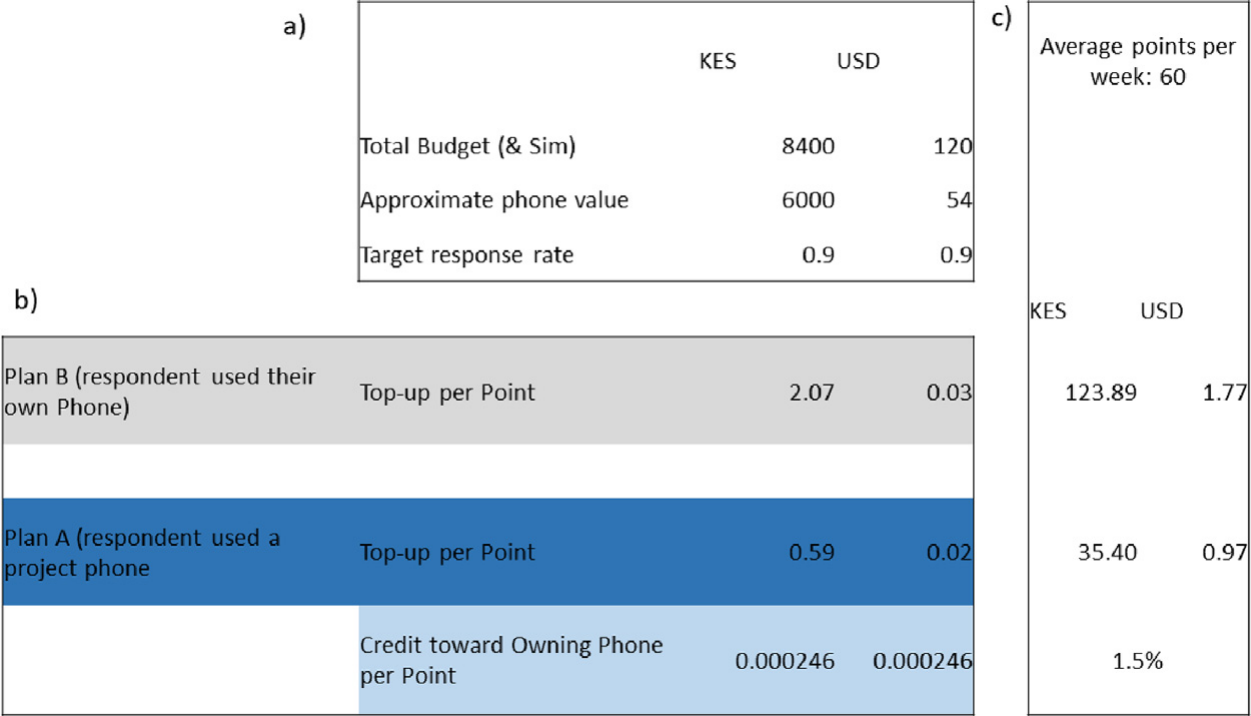
Overview of the points system based on the project budget and the local cost of smartphones. This illustration comes from the Kenyan field site where a) the local cost of a smartphone was approximately 54 USD (6000 Kenyan shillings). The top up value per point b) was dependent on if a respondent used their own smartphone (Plan B) or if they were using a project phone (Plan A). If participants were on plan A, they had a reduced top up per point, but were also gaining credit to owning the phone at the end of the yearlong survey. This ensured the compensation within each country remained fair. In some cases, people chose to use a project phone instead of their own one. On average c) an individual was likely to score approximately 60 points per week, though this may be higher with greater participation and selecting for additional bonus points. With an average weekly score an individual on Plan B might score $1.77 towards data and talk time, however an individual on Plan A might only gain $1 in compensation for 60 points but would gain 1.5 % towards the phone ownership. Each country had their own budget and differing costs of phones.

Where an individual used a project phone, they received a reduced top-up per point (Plan A see Fig. 3), however they also received a percentage of credit towards ownership of that phone. From the example illustrated in Fig. 3, an individual in Kenya may have scored approximately 60 points per week. This score would gain them $1 in phone top ups, but also 1.5 % towards the phone ownership, which they would keep at the end of the project. However, if the respondent used their own phone, they would receive almost $2 per week if completing most tasks. Points calculators per country can be found in Folder 3 in the repository. Note that in south Africa, the budget was increased due to higher data costs (see Table 2 for compensation across the case studies).

**Table 2:** Overview of country, sample size and research length.

Country	Study year	Survey Language	Number of respondents included in final sample.	Total research length (weeks)	Total compensation budget per respondent (including phone costs; USD)	Average weekly response rateα	Attrition rate (%)¥
Kenya	2022/23	Swahili & English	109	80	145	76	73
Peru	2022/23	Spanish	98	80	120	55	56
South Africa	2022/23	Xhosa & English	103	80	325	29	54
Haiti	2022/23	Haitian Creole & French	Unable to continue project after the pilot.				

α Calculated over the key survey period (weeks 18–55).

¥ Calculated as dropping off before cut off point.

### 4.3 Sampling strategy

In every country, a gate keeper was sought to select our sample of 50 CBS users and 50 non users in each country (with a target overall sample of 300 households). In both Kenya and Peru, the gate keeper was the CBS provider. In Cape Town, South Africa two local research assistants who knew the area worked with several community leaders to build a list of CBS households (the City of Cape Town provides free household CBS at scale, but were not engaged as gatekeepers as it was not appropriate in this setting). See Table 2 for an overview of the case study sites.

I. The gate keeper in each country was asked to suggest 150 CBS user households (defined as households with CBS within their property/compound) with a good geographic spread over the study site.
II. From these lists, we randomly selected 50 households to become our CBS user sample. In 25 of these households (randomly selected) we aimed to approach/survey an adult female member of the household, and in the other 25 we approach/survey an adult male.
III. We asked each person in the CBS user sample to identify three nearby households that were similar to them (i.e., similar property/compound [e.g., size, construction material etc.] with a similar number of people in the household, and similar presence/absence of children, within the same study site) but did not have CBS within their property/compound.
IV. From each list of three, we randomly selected one. The gender from the randomly selected nearby household matched that of the CBS user (i.e., so if a female CBS user gave us a list of three households, then we would randomly select one and survey and adult female in that household; and vice versa for males). In the Peru case study, not every CBS user was able to provide further references. In these cases, the CBS organisation was approached to invite more households to participate in the study.
V. The aim was for each country to have a paired list of 50 CBS users and 50 CBS non-users, with an even gender balance with a total sample of 300 across all countries.
VI. Where individuals refused or dropped out within the first 3 months of the study, another individual was added to the sample.
VII. Individuals that did not complete tasks for several weeks were contacted by the researchers and asked if there were technical or other barriers preventing them completing in the project.

Once a respondent had agreed and consented to participate in the project, they were invited to one of a series of training workshops hosted by the field teams. In some cases, individuals were trained 1:1 but often in groups of 10+. The workshops introduced the respondents to the project, explained how the data was to be used, and invited them to do some practice tasks. Some of the tasks included using GPS or selecting multiple choice options. Local research team members were on hand to deal with any technical difficulties. A copy of the training material can be found in the repository. Ongoing technical support workshops were offered by the in-country teams, this was either a regular monthly event (such as in Kenya), regular phone calls, or support over WhatsApp.

### 4.4 Survey design

The survey was designed by specialists in the research team (wellbeing, poverty and shocks by A.L, A.B, S.W, K.C; sanitation related questions by F.A, D.B, M.D, P.H, A.L, H.L A.M, A.O, J.R, A.P). In each country the surveys were double blind translated by members of the research team. In each country a pilot study was conducted to ensure the survey instrument worked and capture any issues with the survey coding or translations. In all cases, small changes were made to the wording of the questions, except where they followed standardised metrics to capture information, such as the World Health Organisation Wellbeing Index, see Table 3 for a summary of each task type and the key references per section. After the pilot stage, the project was not continued in Haiti due to ongoing socio-economic and political instability.

**Table 3:** Overview of the survey elements.

Task name	Frequency	Summary and key references
Metadata	All Tasks	Start and end time logged
Demographics	One off	Contains household roster as well as asking about vulnerabilities within the household using an series of questions from the Washington Group[12].
Poverty	Quarterly	Uses a poverty index from Innovations for Poverty Action[4]
Sanitation Networks	Quarterly	Captures social network of Container Based Sanitation (CBS) users.
WASH (Water, Sanitation and Hygiene)	Weekly, Monthly and Quarterly versions	Uses standardised classifications for toilet systems and water points[6]. Key questions included water sources for drinking, washing and toilets.
Elections and politics	Weekly	Captures sanitation related campaigning.
Shocks	Weekly	Captures if a livelihood or environmental shock was experienced, the shock category (e.g., livelihood, health, or weather) as well as self-reported impacts on the individual.
Sanitation access	Weekly	Uses standardised classifications for toilet systems and water points[6]
Electricity access	Weekly	Captures interruptions to electricity experienced by the household
Wellbeing	Weekly	We used multiple metrics of wellbeing including World health organisation (WHO-5) index[7] as well as sanitation specific wellbeing questions as developed by Ross (2021)[8]
Demographics [Expenditure]	Weekly	Key expenditures by group (e.g., rent, food, travel).

Certain elements of each task were rewarded by “bonus” points, to reduce survey fatigue amongst respondents. Many of the tasks were introduced by questions such as “Has anything changed with [task type such as a new shock, new sanitation access issue] since we last spoke to you?”. This enabled us to use skip logic to move the respondent quickly through the survey. In some cases where questions were skipped these data remain empty (in e.g., Sanitation Access) and have not been filled in during data processing (see the cleaning protocol descriptions below for all processed data). In some cases, e.g., the WASH weekly survey, the file is suffixed with “Filled”, this denotes where the previous entry was carried to the following blank week.

### 4.5 Survey ecosystem

Once the survey questions had been finalised these were converted into ODK format using the xls method [9]. The default language was set at this time, so each country had duplicate forms with either a default in language A or B (this was country specific; see Fig. 1). To account for any lag time in training the 100+ people per country we allowed the survey to run for 80 weeks (to ensure we captured 52 weeks per respondent). In some instances, people were trained over 1 month apart. Once someone had completed a year, they did not need to complete any further tasks. The server was closed after 80 weeks.

In total 3022 forms were created and hosted on the ODK Cloud server [9]. We had 14 different user types (for example, a container-based sanitation user that spoke Swahili was able to access only the forms available to that specific User type; Kenya_Swahili_CBS). Excluding the forms developed for the Haiti project, over 560 forms were available to download to each respondent.

**Fig. 4:**
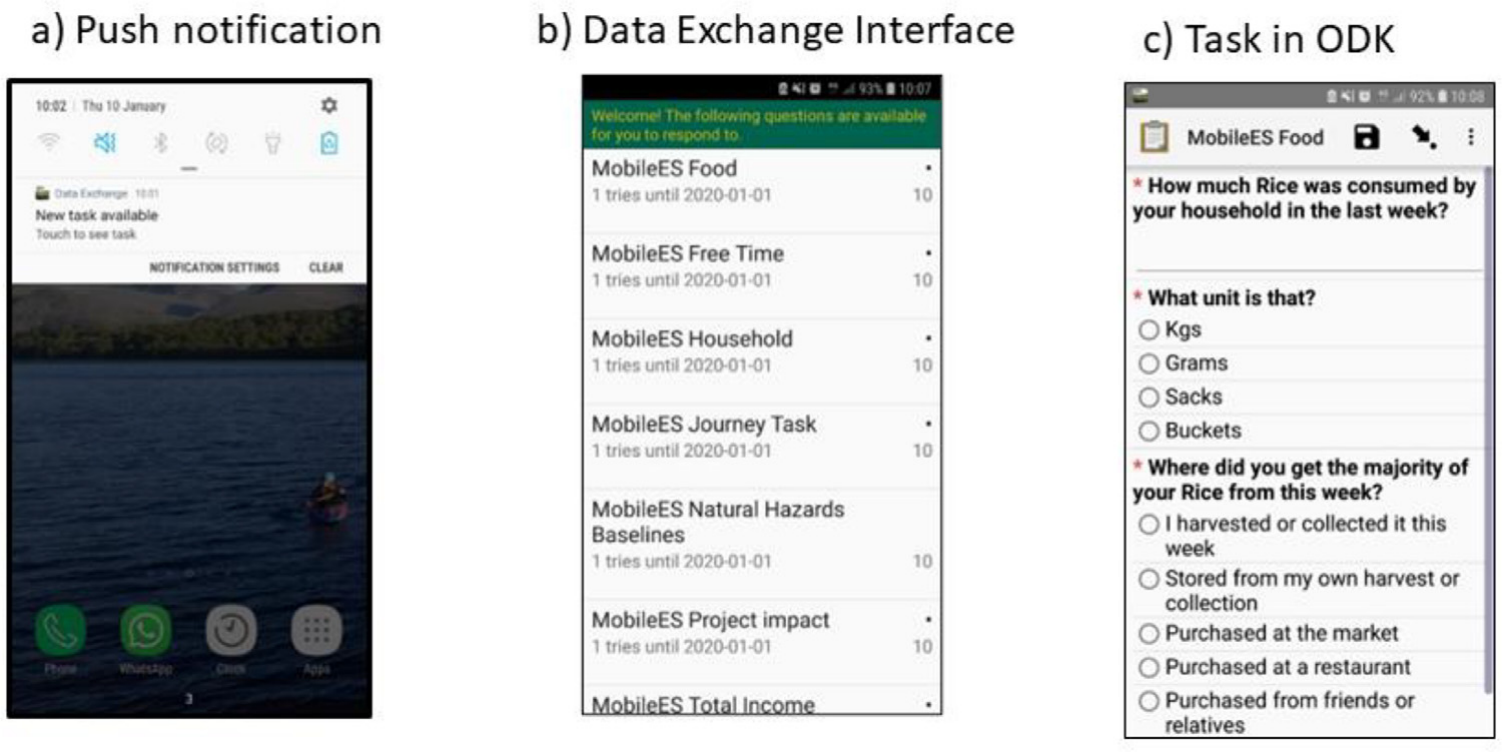
The Data exchange app allows push notifications to nudge participants to complete a task soon after it becomes available (a). The Data exchange interface (b) gives an overview of the task topic, its value in points and how long it is available for. Once a participant has selected a task, they are then taken to the correct ODK form to fill (C).

The survey was designed so that there were only five weekly tasks arriving on a Monday-Friday with a weeklong expiration date. Once per month there was an additional 1–2 tasks. The ODK form allows a separate csv file to be read, which is stored as a separate media file to the main survey (See Table 4 for the code from these surveys). For each form we had a separate csv containing the form's points value as well as its expiration date. Weekly tasks were available for seven calendar days. The bespoke Data Exchange app [2] enabled us to create a user-friendly interface where an individual was not faced with over 500 forms to complete (and we wanted them to complete them evenly throughout the year). The app also reads the external csv file which allows it to “nudge” participants by saying “new form available today”, or “task expiring today” (see Fig. 4). A full list of all tasks in each country with their start, expiration date and points value are available in the depository.

**Table 4:** ODK code allowing a separate media csv file to be read external to the main survey.

type	name	label::English (en)	calculation
calculate	start_date	start_date	pulldata('prefill','start_date','key',${key})
calculate	expiration_date	expiration_date	pulldata('prefill','expiration_date','key',${key})
calculate	max_submissions	max_submissions	pulldata('prefill','max_submissions','key',${key})
calculate	task_value	task_value	pulldata('prefill','task_value','key',${key})
calculate	task_length	task_length	pulldata('prefill','task_length','key',${key})

### 4.6 Preprocessing of the data

Below are the key steps for the data cleaning and transformation of the final data sets.

I. Data was stored on ODK Central server encrypted. This has since been removed and deleted.
II. Each week data was downloaded using RUODK package [1]
III. Empty files were identified and removed. This often occurred in the first and last weeks of the project.
IV. Data was merged based on task type, and form name was added. This enabled us to create a distinction of weeks.
V. Variables that were identified as not required for early analysis were redacted.
  a) This was using redact function in R (numbers changed to 9, and text to [redacted])
  b) Phone numbers and household locations removed.
VI. Data was appended to the ID KEY, and entries with no match were removed- this accounts for enumerators practicing, early trials, and those filling it in who did not consent to be part of our study.

**Protocol description for Demographics baseline**

I. Appended the Demographics baseline file for the three countries.
II. Identified any duplicates on ID and keep the earliest entry based on end date – this accounts for people submitting the demographics baseline multiple times. We decided to keep the earliest entry, as this is the entry they completed under supervision in workshops with the teams.
III. Once we had a final document without duplicates, we checked the user type and the gender for each ID against the information from the consent forms, which had been provided and checked by the local teams. In case of any discrepancies in either gender or user type, we kept the information from the consent form and assumed there was an input error by the participant. In extreme cases where an ID had a specific gender in the consent form, but they repeatedly selected a different gender in the demographics baseline, we double-checked the information with the local team.
IV. We also checked the age of the participants. For Kenya and Peru, age was not supplied in the consent forms. Therefore, for each ID, we checked the age column for all the entries from the demographics baseline. If all the entries had same/similar ages, we kept the first entry – this accounts for people who are unsure about their age. If entries were very different, but there was a particular age that was clearly more frequent than the age stated in the first entry, we kept the most frequent age and assumed there was an input error by the participant. In cases where we were unsure, we checked the information with the local teams.
V. For South Africa, as the consent forms included the age, we kept the age from the consent form in case of discrepancies.
VI. Once all the discrepancies were corrected, we created a final master file. This file is to be used as the baseline to cross-reference questions in the other surveys – for example, in cases where we asked questions that should only be answered by CBS users, we use the demographics master file to remove any non-users who might have answered those questions by mistake. Similarly, in the surveys where we asked about menstrual health management, we use the demographics master file as a reference to remove any male participants who might have answered those questions.

### 4.7 Protocol description for the rest of the surveys

**Fig. 5:**
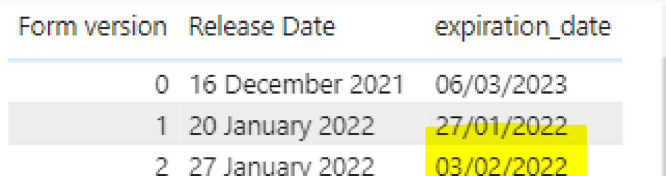
In some cases, form release dates were overlapped. The final form versions assign the forms to a specific date.

I. Appended the data for all the countries.
II. Identified any duplicates on ID+Form version and kept the earliest entry based on end date – this accounts for people submitting forms multiple times when they do not receive a confirmation message immediately.
III. Found all the out-of-range/invalid entries:
  a. The start time was after/on the release date (start_date in the survey) AND before/on the expiration date AND
  b. The end time was before/on the expiration date.
IV. Identified whether the out-of-range entries fit within the active period for any other form version (excluding the baseline), where the active period is defined as any date between the release date and the expiration date for a survey, both inclusive. Note that active periods for the same form version might be different for different countries.
V. To do this, we checked whether the start time and end time of that entry are within the release date-expiration date range for any other form version, and checked whether there is an existing valid entry for that other form version - we only assigned a new form version number to the out of range entries if there were no valid entries for that new form version in the survey already. This accounts for people filling in the wrong form version in a particular week, but still filling in the survey, so it is still a valid entry, as long as they did not fill in two surveys on the same week.
VI. Removed any duplicates in the out-of-range entries where for the same ID and Form Version we have assigned more than one potential new form version. This accounts for situations where an entry could fit under two different form versions because there is an overlap in dates in the forms (e.g. in the example shown below, if we have a submission for form version 1 but it was filled in on the 3rd of Feb, it is out of range for Form 1 but it could fit under Form 2; if there are no valid entries in the survey for Form 2, the cleaning protocol will assign Form 2 to the out of range entry.
VII. Removed any duplicates in the out-of-range entries where the same new form version has been assigned to different ID&Form version combinations. E.g., This accounts for people filling in multiple surveys in the same day. For example, if someone filled in surveys 2, 3, 4, and 5 on the same day, and that day corresponds to the timeframe for form version 10, we would only keep the entry for form 2 and would reassign it to form 10, the other entries would be removed.
VIII. If a match is found in 4–6, correct the form version, release date and expiry date, and correct the status of the entry from out of range/invalid to valid/in range.
IX. Filter out all the invalid entries (Fig. 5).

### 4.8 Detail of the data

**Fig. 6:**
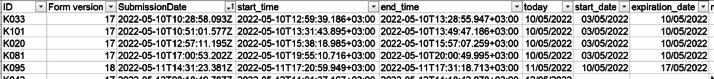
Screen shot of the columns found in the data set.

The screenshot below (Fig. 6.) shows an example of the columns found in the dataset:

The columns used for the cleaning protocol are described below:

I. Form version corresponds to the week number when participants should fill in the form.
II. SubmissionDate is the date when the survey reached the server. In cases when participants have internet connection issues or they do not have any money available in their phones, these dates may have a delay with respect to when participants completed the survey. The data cleaning protocol accounts for this.
III. Start_time and end_time corresponds to the times when participants started and finished filling up the survey.
IV. start_date is the date when the survey was released – referred to as “release date” in this document. The survey should not be available before this date.
V. Expiration_date is the date when the survey closes. The survey should not be filled in after this date.
VI. The data on the repository has been filled in with regards to the skip logic for the WASH Weekly survey, using data from the previous entry. For example, in many cases at the beginning of the survey we asked respondents if “anything” had changed since last week. If the response was “no change”, this data set has duplicated the data from the previous week[s].

## Limitations

**Smartphone Compatibility Challenges:**

• Increased smartphone prevalence does not always correspond with smartphones that are compatible with the smartphone application.
• ODK's Android exclusivity and varying capabilities of Android phones pose obstacles.
• Outdated Android systems and device-specific issues impact functionality in Peru and South Africa.

**Testing and Technical Support:**

• Extensive testing of smartphone handsets is crucial before distribution.
• Cost disparities and Android updates necessitate constant adaptation and technical support.
• Remote access software like Anydesk [11] proves effective, but data costs influence its usage.

**Survey encountered setbacks due to software evolution and updates.**

• Continuous software development is a dynamic process and required multiple updates for the data exchange app since it was first built in 2015.
• Future studies may consider ODK-X's customizable interfaces and bi-directional synchronization offers enhanced capabilities for data collection.

### Sampling bias

• The smartphone access rates within countries vary, which means there are potential biases in our sample towards literate individuals. Generalizations to wider populations should consider this bias.
• Barriers such as literacy, numeracy, and technological proficiency persist, hindering participation in smartphone-based surveys. However, these barriers are gradually diminishing with the increasing prevalence of smartphones in everyday life across urban and rural areas over time [12].

## Ethics Statement

In each country local permission was sought by the relevant bodies. Ethical approval for the project was approved by Bangor university, UK, College of Environmental Sciences and Engineering Ethics Committee Approval Number: COESE2021SW01A. Team leads in all countries sought ethical approval from relevant bodies (see the SOS_Permits folder in repository 1) and informed consent was sought from all survey participants to take part in the studies. An example consent form is available along with the reshare data depository.

## CRediT authorship contribution statement

**Amy R. Lewis:** Conceptualization, Investigation, Methodology, Software, Formal analysis, Validation, Data curation, Writing – original draft, Writing – review & editing. **Andrew R. Bell:** Conceptualization, Investigation, Methodology, Software, Formal analysis, Validation, Data curation, Writing – review & editing, Funding acquisition. **Ana Casas:** Methodology, Software, Formal analysis, Validation, Data curation, Writing – review & editing. **Beata Kupiec-Teahan:** Conceptualization, Formal analysis, Validation, Data curation, Writing – review & editing. **José Mendoza Sanchez:** Conceptualization, Formal analysis, Validation, Data curation, Writing – review & editing. **Simon Willcock:** Conceptualization, Investigation, Methodology, Software, Formal analysis, Validation, Data curation, Writing – original draft, Writing – review & editing, Funding acquisition. **Fiona Anciano:** Conceptualization, Methodology, Writing – review & editing, Funding acquisition. **Dani J. Barrington:** Conceptualization, Methodology, Writing – review & editing, Funding acquisition. **Mmeli Dube:** Conceptualization, Investigation, Methodology, Writing – review & editing. **Paul Hutchings:** Conceptualization, Methodology, Writing – review & editing, Funding acquisition. **Caroline Karani:** Investigation, Investigation, Writing – review & editing. **Arturo Llaxacondor:** Methodology, Investigation, Writing – review & editing. **Hellen López:** Conceptualization, Investigation, Methodology, Writing – review & editing, Funding acquisition. **Anna L. Mdee:** Conceptualization, Writing – review & editing, Funding acquisition. **Alesia D. Ofori:** Conceptualization, Writing – review & editing. **Joy N. Riungu:** Conceptualization, Writing – review & editing, Funding acquisition. **Kory C. Russel:** Conceptualization, Writing – review & editing, Funding acquisition. **Alison H. Parker:** Conceptualization, Writing – review & editing, Supervision, Project administration, Funding acquisition.

## Declaration of Competing Interest

The authors declare that they have no known competing financial interests or personal relationships that could have appeared to influence the work reported in this paper.

## Data Availability

Longitudinal Sanitation Data From High-Frequency Phone Surveys Across Three Countries, 2020–2024 (Original data) (ReShare)

## References

[1] Lewis, A.R. et al. Longitudinal sanitation data from high-frequency phone surveys across three countries, 2020–2024. https://reshare.ukdataservice.ac.uk/857073/(2024). 10.1016/j.dib.2024.110635 PMC11259941 39035842

[2] MobilES – msds.tools.

[3] CBSA. About container based sanitation – container based sanitation alliance. https://cbsa.global/about-cbs (2024).

[4] Renaud F.G., Birkmann J., Damm M., Gallopín G.C. (2010). Understanding multiple thresholds of coupled social–ecological systems exposed to natural hazards as external shocks. Nat. Hazards.

[5] IPA. IPA Poverty probability Index. *PPI* https://www.povertyindex.org/ppi-country.

[6] WHO. Core questions | JMP. https://washdata.org/monitoring/methods/core-questions (2024).

[7] WHO. WHO-5 Questionnaires. https://www.psykiatri-regionh.dk/who-5/who-5-questionnaires/Pages/default.aspx (2024).

[8] Ross I. (1982). How does sanitation influence people's quality of life? Qualitative research in low-income areas of Maputo, Mozambique. Soc. Sci. Med..

[9] ODK. ODK - Collect data anywhere. https://getodk.org (2023).

[10] Mayer, F.W. ruODK: an R Client for the ODK Central API. Zenodo 10.5281/zenodo.5559164 (2021).

[11] AnyDesk. AnyDesk The Fast Remote Desktop Application. *AnyDesk* https://anydesk.com/en.

[12] ITU Data Hub. Mobile-cellular subscriptions - ITU DataHub. *Mobile-cellular subscriptions* https://datahub.itu.int/data/?c=701&i=178&u=per+100+people&e=ZAF.

